# Tunable Transparent Conductors Based on SnO_2_: Theoretical and Experimental Studies of Codoping

**DOI:** 10.1021/acsomega.4c07860

**Published:** 2024-12-04

**Authors:** Wenjing Qian, Xianghui Feng, Yanxue Wang, Ahmet Nazligul, Yiwen Lu, Mingqing Wang, Wei Wu, Kwang Leong Choy

**Affiliations:** UCL Institute for Materials Discovery, University College London, Malet Place, London WC1E 7JE, United Kingdom

## Abstract

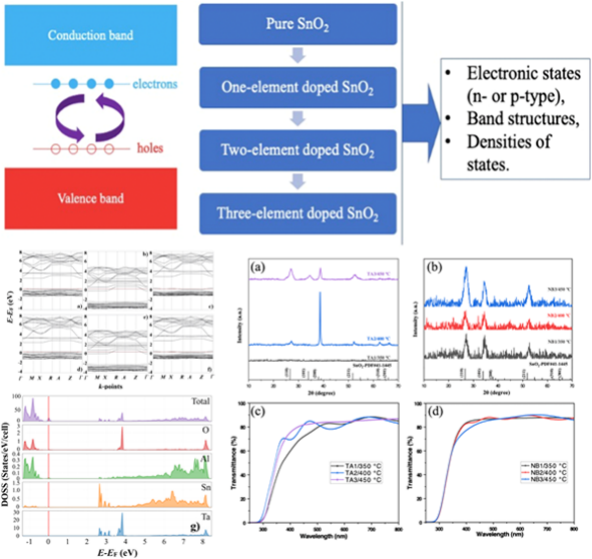

Transparent conducting oxides (TCOs) are widely used in modern electronics because they have both high transmittance and good conductivity, which is beneficial for many applications such as light-emitting diodes. Tailoring electronic states and hence the conductive types by design is important for developing new materials with optimal properties for TCOs. SnO_2_, with a wide band gap, low cost, no toxins, and high stability, is a promising host material for TCOs. Here, we performed a set of hybrid-exchange density functional theory calculations on the two-element and three-element codoped SnO_2_ by using Sr, Ta, Al, Ga, V, and Nb, which were then validated by the relevant experimental works on SnO_2_. As predicted by the first-principles calculations, the controllability of the electronic states to be n- or p-type can be demonstrated experimentally by varying the relative doping concentration between donors (Ta/Nb) and acceptors (Al/Ga). One of the main advantages for these codoping methods is that the charge neutrality problem caused by the dopant can be circumvented. The thin films fabricated showed a low sheet resistance (down to ∼450 Ω**/□**) and a high optical transparency (above 80%). The combination of our calculations and experimental material fabrication and characterizations has shown a great potential for codoping SnO_2_ for (i) the efficient processing of the integrated circuit composed of both p-type and n-type transistors (using the same target precursors during the deposition) and (ii) a good lattice matching for p–n junctions. Most importantly, our calculations, supported by the experimental works, point to a promising route to accelerate the discovery process for the alternative cost-effective and high-performance indium-free TCOs using computational material design.

## Introduction

Tailoring the electronic state by using dopants to be either electron- (n-type) or hole transport- (p-type) type is the central theme of semiconductor physics. Transparent conducting oxides (TCOs) are not only crucial for optoelectronic devices but are also promising for many other applications such as flexible electronics because they can exhibit the duality of an excellent electrical conductivity and a high optical transparency.^1^ The conductivity of TCOs could be tuned to be n-type (p-type) through doping electrons (holes). With the sheet resistance reaching down to a few Ω**/□** and the transparency exceeding 80%, TCOs have attracted great interest for application in displays,^2^ photovoltaics,^3^ and light-emitting diodes (LEDs).^4^ Through different processing methods, TCOs can be fabricated into single crystals, thin films, and nanostructures. One example for this is the latest flexible molybdenum-doped indium oxide (IMO)^5^ film with a sheet resistance down to 1.37 Ω**/□** corresponding to a mobility as high as 109 cm^2^ V^–1^ s^–1^. Moreover, by introducing appropriate dopants, TCOs can be manipulated into the n- or p-type to form p–n junctions.^6^ Currently, the commercially available n-type TCOs are mostly fabricated by doping the post-transition metal oxides,^7^ such as In_2_O_3_, SnO_2_, and ZnO. However, the lack of p-type counterparts with a comparable performance is still limiting the application of TCOs. In addition, it would be ideal to be able to control the type of conductivity for TCOs.

While ITO (Sn-doped In_2_O_3_) has dominated the TCO market for decades, the scarcity of indium significantly elevated the cost of ITO and restricted the further development of high-performance TCOs.^1^ Potential alternative candidates for TCOs have been investigated intensively in recent years. Among these, tin oxide (SnO_2_) is a semiconducting material with a band gap of ∼3.6 eV, similar to that of In_2_O_3_ (3.75 eV).^8^ The low price, ease of doping, excellent conductivity, and optical transparency make SnO_2_ competitive and thus promising for mass production. Many elements have been exploited to dope SnO_2_ to render the n-type conductivity, including fluorine,^9^ nitrogen,^10^ phosphorus,^11^ antimony,^12^ tantalum,^13^ niobium,^14^ and vanadium.^15^ However, very few metallic ions have been proven to be the suitable p-type dopants for SnO_2_, such as gallium.^16^ The aluminum- and strontium-doped SnO_2_ materials have exhibited both the characteristics for the p-type^17^ and the n-type,^18^ which is controversial. Furthermore, the research mentioned above suggests that there might be controllability on the electronic state between n- and p-types for these types of materials.

Recently, the carrier mobility in F-doped SnO_2_ (FTO) was reported to be limited by the preference of the F cation to be incorporated into the interstitial positions at higher doping concentrations.^19^ In the case of Sb-doped SnO_2_ (ATO), the formation of Sb^3+^ ions could trap electrons, thus decreasing the mobility.^20^ Subsequently, Williamson et al.^21^ proposed that an ideal n-type dopant should be stable at a valent state and would not hybridize with the Sn *s* state. Following this hypothesis, Ta, Nb, and V are the three promising candidates for the n-type dopants. More specifically, Ta-doped SnO_2_^13^ has been experimentally proven to exhibit a sheet resistance down to <10 Ω**/□** (corresponding to a mobility of 80 cm^2^ V^–1^ s^–1^), surpassing FTO/ATO. Nb doping^14^ has also led to a sheet resistance of 30 Ω**/□** (a mobility of 23.9 cm^2^ V^–1^ s^–1^). However, since Ta, Nb, and V are relatively new dopant candidates, they have not been included in the codoping studies for SnO_2_ before, which inspired us to investigate these three elements. As compared with n-type doped SnO_2_, generating the p-type doping is more challenging due to the strong electronegativity of O^2–^ anions that can be found as the native defect. To address this issue, an approach of codoping the shallow acceptors with donors has been taken into consideration in the previous work. For instance, Ga–N codoped SnO_2_^22^ witnessed a n- to p-type transition experimentally with a sheet resistance of ∼3 × 10^4^ Ω/□ (∼3 order of magnitude higher than ITO) and so did S–Al codoped SnO_2_.^23^ The resulting electrical properties are approximately in the order of 10^4^ Ω/□ for S–Al codoped SnO_2_, incomparable with metallic-element-doped SnO_2._^24^ Previously the (Sb, Zn)^25^ pair has also been codoped into SnO_2_ experimentally to enhance the densities of ceramics, in which the authors found that codoped SnO_2_ was a n-type semiconductor with a sheet resistance of ∼3 × 10^3^ Ω/□, corresponding to a resistivity down to 0.17 Ω cm, two orders higher than Sb-doped SnO_2_. In addition, the (Fe, Al)^26^ pair has been mixed in the SnO_2_ nanoparticles experimentally to facilitate the integration of optoelectronics and spintronics, taking the advantages of the magnetic properties and conductivities due to the doping of Al and Fe. Moreover, La–S codoped SnO_2_^27^ has been studied theoretically using density functional theory (DFT) at the level of the generalized gradient approximation (GGA). The results therein suggested a potential enhancement in the electrical conductivity because increasing the doping concentration of La and S can improve the orbital hybridization.

In this work, we have (i) theoretically investigated codoped SnO_2_ with two or three metallic elements using hybrid-exchange DFT and (ii) experimentally fabricated and characterized the electrical and optical properties of codoped SnO_2_. Owing to the aforementioned high carrier mobility of both Ta- and Nb-doped SnO_2_, Ta, Nb, and V have been chosen to play as the electron donors in our work, where V is in the same group family with Ta and Nb. Meanwhile, Sr, Al, and Ga have been introduced as the electron acceptors in the codoping pairs. These metallic elements have never been theoretically investigated in the codoping pairs in SnO_2_. In parallel, we have performed the corresponding experiments to fabricate the n-type and the p-type TCOs based on the codoping of Ta/Nb and Ga/Al. Our combined theoretical and experimental works revealed that the electronic states can be tuned by codoping different metallic elements, with excellent optical transparency and sheet resistance. In addition, we have observed the half-metal characteristic in (Ta, Sr, V) three-element codoped SnO_2_ from our calculations. This will, therefore, open up a new route to manipulate the electronic state of TCOs by optimally codoping SnO_2_.

## Computational and Experimental Methods

The calculations of the band structures, electronic properties, and wave functions for doped SnO_2_ were carried out by DFT implemented in CRYSTAL14.^28^ The advantage of the CRYSTAL code includes a Gaussian-based localized basis set and an efficient algorithm to screen the time-consuming two-electron integrals, which can provide highly accurate results. The band structure has been plotted along the high-symmetry points of the first Brillouin zone for the rutile structure as G (Γ)—M (1/2,1/2,0)—X (0,1/2,0)—R (0,1/2,1/2)—A (1/2,1/2,1/2)—G (Γ). The generalized gradient approximation (GGA) and hybrid-exchange functionals^29^ were used for a comparison, including PWGGA, PBE0, B3LYP, and HSE06.^28^ Among these functionals, PWGGA only takes into account the gradient of the charge densities as a variable to improve the density functional. PBE0, B3LYP, and HSE06 are the so-called hybrid functionals that combine the GGA functional with a proportion of the Hartree–Fock exact exchange to balance the localization and delocalization of the electron wave functions. By comparing with the previous experimental works, the band structures of doped SnO_2_ computed by PBE0 have been found to be more consistent with the experiments; thus, the PBE0 results will be employed for further analysis.

**Figure 1:**
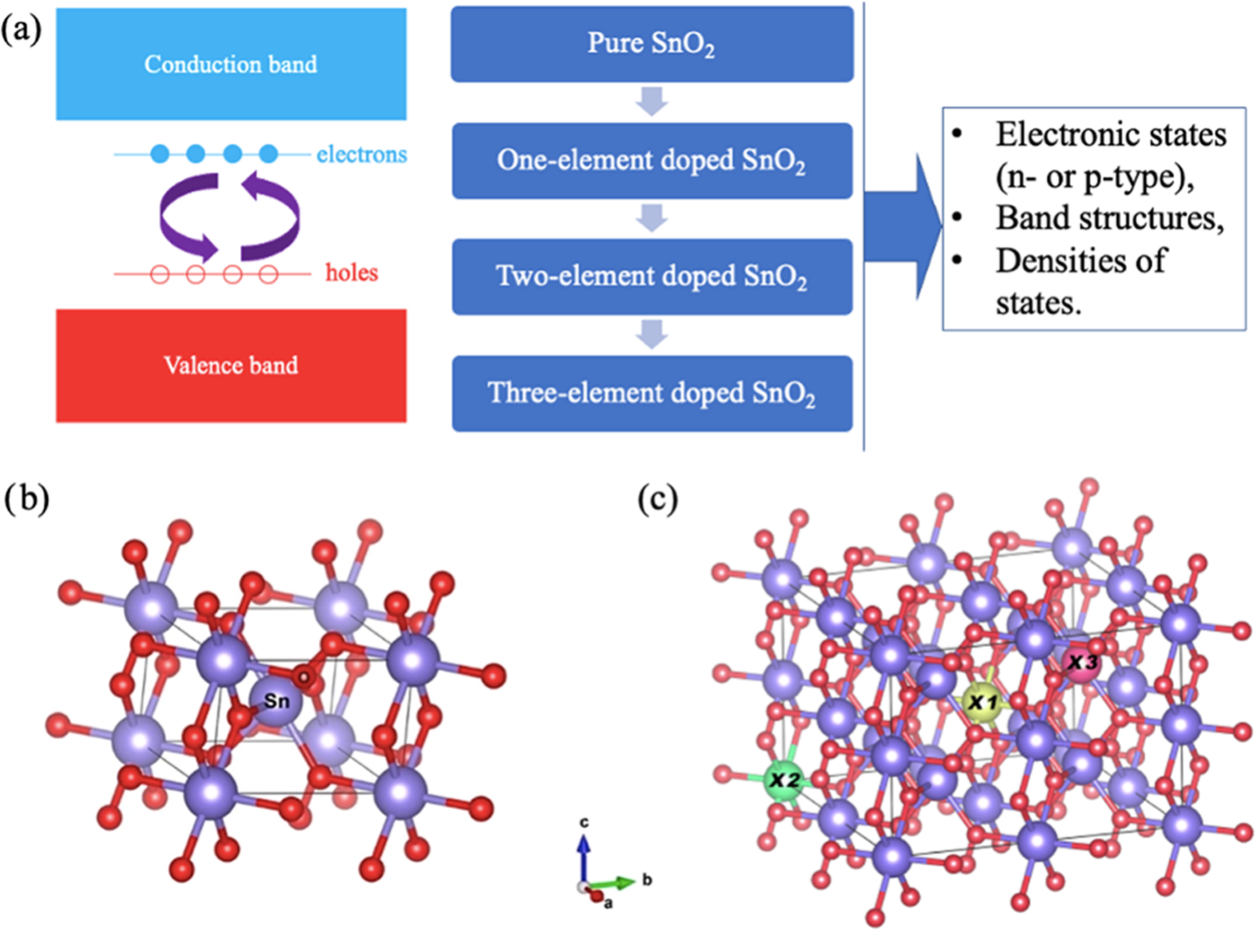
(a) Modeling workflow of this work; (b) the unit cell of rutile SnO_2_ (oxygen atoms in red and tin atoms in purple); and (c) the supercell structure (2 × 2 × 2) of SnO_2_ with doping positions at X1, X2, and X3 used in all calculations presented here.

The rutile SnO_2_ structure (the unit cell is shown in Figure 1b) has a symmetry of the *P*_42_/*mnm* space group, with *a* = *b* = 4.738 Å and *c* = 3.187 Å.^30^ After the geometry optimization by PBE0, the new SnO_2_ lattice parameters (*a* = *b =* 4.826 Å and *c* = 3.265 Å) were used for the simulation. A 2 × 2 × 2 supercell was employed to accommodate the dopant throughout all of the calculations. Please note that the term “cell” will be used to refer to this supercell in the following discussion. The self-consistent convergence energy was set to 10^–4^ Hartree, and a 3 × 3 × 5 Brillouin zone k-point grid was used. As demonstrated in Figure 1c, in the supercell of SnO_2_, the Sn atoms at X1, X2, and X3 positions were replaced by the dopants including Ta, Nb, V, Sr, Al, and Ga. The Fermi level is set to be zero energy in the band structure. As demonstrated in the Figure 1a workflow, pure SnO_2_ was modeled first as a benchmark with the reference to the band gap, as shown in Figure S1 of the Supporting Information (SI). Our calculations indicated a direct band gap of 3.05 eV, which is slightly smaller than the experimental result (∼3.6 eV).^8^ However, the difference would not interfere with the analysis of the doped system because our calculations of Ta-doped SnO_2_ show that it is n-type, which is consistent with the previous experimental results and calculations.^13^ Subsequently, by codoping potential donors (e.g., Ta, Nb, V) with possible acceptors (e.g., Sr, Al, Ga), and changing their doping concentration, the resultant electronic states, band structure, and density of states (DOS) have been generated and analyzed.

Table 1 lists the composition of the SnO_2_ precursors and thin films investigated in this report. As a proof of concept, we deposited the codoped SnO_2_ (Ta/Al, Nb/Al, Nb/Ga, and Nb/Al) thin films and characterized their optical and electrical properties. To optimize the deposition condition of doped SnO_2_ thin films, the films were deposited at three substrate temperatures of 350, 400, and 450 °C. The doped SnO_2_ film was deposited by the aerosol-assisted chemical vapor deposition method.^8,31^ Metal salts (tin(II) chloride, tantalum(V) chloride, niobium pentachloride, strontium nitrate, and the vanadium(III) chloride tetrahydrofuran complex (1:3)) were dispersed in a mixed solvent containing 40 mL of IPA + 8 mL of H_2_O + 2 mL of HCl.^32−35^ The surface morphology and microstructure of oxide thin films were examined using a scanning electron microscope (SEM, ZEISS EVO LS15) equipped with an energy-dispersive X-ray (EDX) spectrometer from Oxford Instruments. The surface roughness of the films in this study was analyzed using a Nanosurf atomic force microscope (AFM) under the tapping mode. The film thickness was measured by the Dektak Stylus profiler (Bruker) with a maximum vertical resolution of 1 Å when using the 6.55 μm range. Please see Figures S7–S9 for more details on the EDX and AFM measurements. The phases and crystal structures of the doped SnO_2_ thin films were determined using an Aeris X-ray diffractometer (XRD, Malvern Panalytical, U.K.) with Cu Kα radiation, spanning a 2θ angle ranging from 10 to 70° at 40 kV and 7.5 mA. The optical transmittance spectra of TCOs were captured using the ultraviolet–visible–near-infrared (UV–vis–NIR) spectrometer (PerkinElmer Lambda 500) within the wavelength ranges of 250 to 800 nm. The Hall effect, in which the voltage difference is generated by the Lorentz force in an electrical conductor situated in a magnetic field, is used to determine whether electrons (n-type) or holes (p-type) are responsible for the electrical conductivity. The Seebeck effect, employed to evaluate the conductive properties of films, is observed when a temperature gradient is present across the film. The Seebeck effect of TCO films was measured using Linseis LSR-1.

**Table 1:** Composition of the Doped SnO_2_ Precursor and Thin Films Deposited at 400 °C (Atomic Percentage)

	precursors			thin films		
TA1	Ta (2.1%)	Al (4.2%)	Sn 93.7%	Ta (3.3%)	Al (6.4%)	Sn 90.3%
TA2	Ta (4.2%)	Al (2.1%)	Sn 93.7%	Ta (3.4%)	Al (6.6%)	Sn 90.0%
NB	Nb (2.1%)	Al (4.2%)	Sn 93.7%	Nb (3.2%)	Al (6.2%)	Sn 90.6%
NG	Nb (4.2%)	Ga (2.1%)	Sn 93.7%	Nb (6.8%)	Ga (2.9%)	Sn 90.3%
TG	Ta (2.1%)	Ga (4.2%)	Sn 93.7%	Ta (6.6%)	Ga (3.0%)	Sn 90.4%

## Results and Discussion

**Figure 2:**
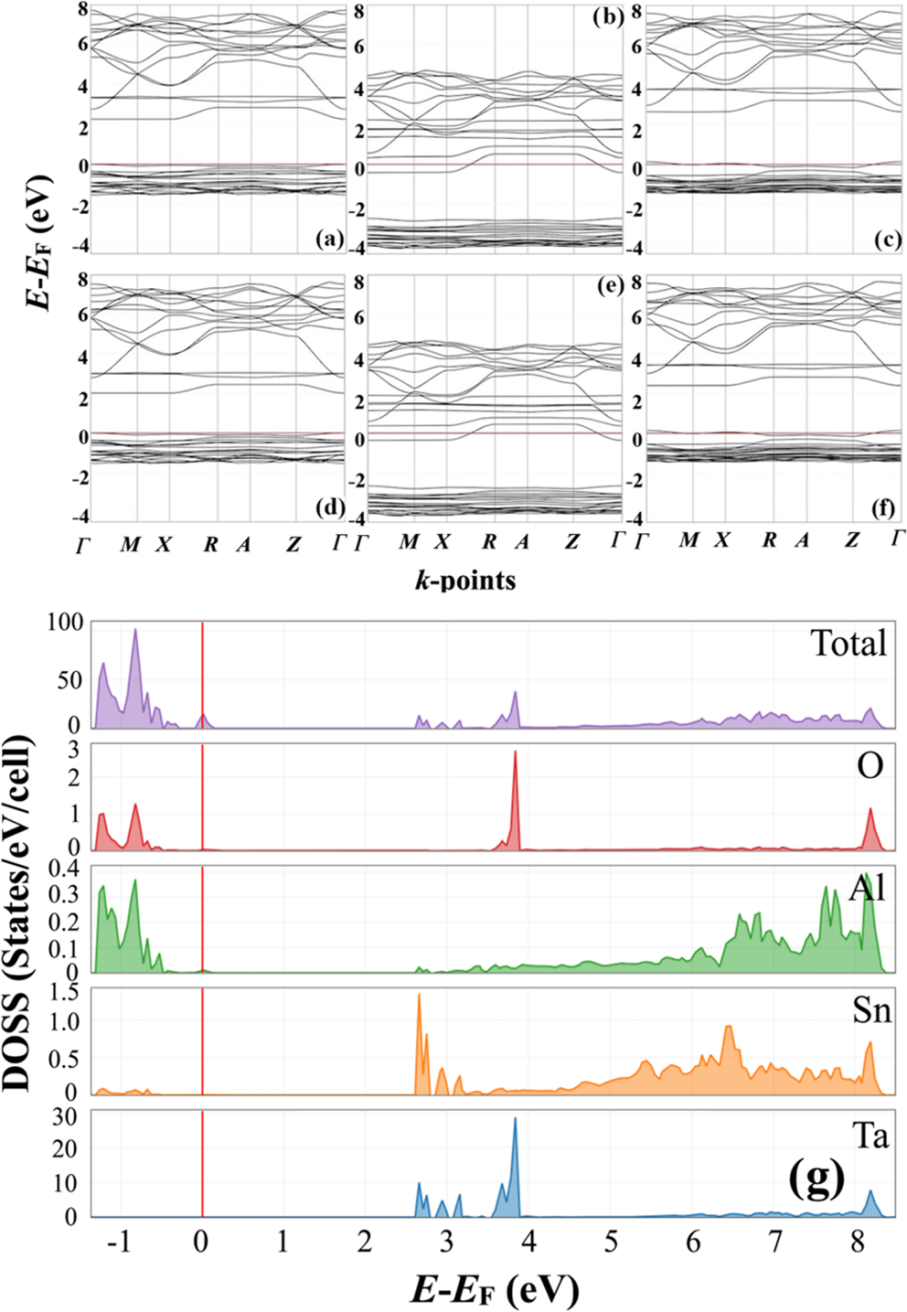
Electronic structures of codoped SnO_2_ with (a) one Ta atom/cell and one Al atom/cell; (b) two Ta atoms/cell and one Al atom/cell; (c) one Ta atom/cell and two Al atoms/cell; (d) one Nb atom/cell and one Al atom/cell; (e) two Nb atoms/cell and one Al atom/cell; and (f) one Nb atom/cell and two Al atoms/cell. (g) DOS corresponding to the band structure calculation in panel (c).

As shown in Figure 2, we can see the tuning process for doping n- and p-type dopants (Ta/Nb and Al, respectively). The codoping of 1 Ta atom/cell and 1 Al atom/cell would lead to an insulating state, 2 Ta atoms/cell and 1 Al atom/cell to the n-type electronic state, and 1 Ta atom/cell and 2 Al atoms/cell to the p-type electronic state. These computational results suggest that the electronic state of SnO_2_ could be manipulated using Ta and Al with different concentrations, pointing to the route to achieve a bespoke electronic state by tailoring the relative concentrations of the codoping chemical elements. Figure 2g shows the DOS for codoping with 1 Ta atom/cell and 2 Al atoms/cell, which would allow us to analyze the contributions of different atoms at different energy regions. The main contribution at the Fermi level is from the Al atom, whereas the conduction band minimum (CBM) is dominated by Sn and Ta, as expected for the hole (Al) and electron (Ta) dopants.

In addition, we have performed the similar calculations for the other two-element codoped SnO_2_, as summarized in Table 2. Please see more details for the computed electronic structures in Figures S1–S6. The other pairs of the doped elements except (Ta, Al) and (Nb, Al), including (Ta, Sr), (Nb, Sr), (Ta, Ga), and (Nb, Ga), have been investigated. In these calculations, we can see that doping with (Ta, Al), (Nb, Al), (Ta, Ga), and (Nb, Ga) can provide the electronic-state manipulations as we expected between the n- or p-type semiconducting materials. On the other hand, the (Ta, Sr) and (Nb, Sr) pairs within the atomic ratios investigated in this paper have presented the p-type behavior (please see Figures S2 and S3). As shown in Figure S3, the Fermi energy of the Ta–Sr codoping is below the VBM by ∼0.32 eV, whereas the Fermi energy for the Ta–Al and Ta–Ga codoping is below the VBM by ∼0.16 eV. This implies that Sr could provide more holes if codoped with Ta than with Al and Ga, which might be due to the more loosely bonded 5s electrons in Sr.

**Table 2:** Two-Element Codoped Groups and Concentrations of SnO_2_

codoping groups	dopants in the supercell	doping concentrations (atom %)	electronic states
Ta and Sr	1 Ta + 1 Sr	Ta (2.1%), Sr (2.1%)	p-type conducting
	2 Ta + 1 Sr	Ta (4.2%), Sr (2.1%)	semiconducting
	1 Ta + 2 Sr	Ta (2.1%), Sr (4.2%)	p-type conducting
Nb and Sr	1 Nb + 1 Sr	Nb (2.1%), Sr (2.1%)	p-type conducting
	2 Nb + 1 Sr	Nb (4.2%), Sr (2.1%)	semiconducting
	1 Nb + 2 Sr	Nb (2.1%), Sr (4.2%)	p-type conducting
Ta and Al	1 Ta + 1 Al	Ta (2.1%), Al (2.1%)	semiconducting
	1 Ta + 2 Al	Ta (2.1%), Al (4.2%)	p-type conducting
	2 Ta + 1 Al	Ta (4.2%), Al (2.1%)	n-type conducting
Nb and Al	1 Nb + 1 Al	Nb (2.1%), Al (2.1%)	semiconducting
	1 Nb + 2 Al	Nb (2.1%), Al (4.2%)	p-type conducting
	2 Nb + 1 Al	Nb (4.2%), Al (2.1%)	n-type conducting
Ta and Ga	1 Ta + 1 Ga	Ta (2.1%), Ga (2.1%)	semiconducting
	1 Ta + 2 Ga	Ta (2.1%), Ga (4.2%)	p-type conducting
	2 Ta + 1 Ga	Ta (4.2%), Ga (2.1%)	n-type conducting
Nb and Ga	1 Nb + 1 Ga	Nb (2.1%), Ga (2.1%)	semiconducting
	1 Nb + 2 Ga	Nb (2.1%), Ga (4.2%)	p-type conducting
	2 Nb + 1 Ga	Nb (4.2%), Ga (2.1%)	n-type conducting

While codoping these elements in SnO_2_ has never been reported before, our results demonstrated the consistency with their single doping cases. To be more specific, Ta-singly-doped SnO_2_ (not shown) exhibited a superior n-type conductivity and a carrier mobility comparable to FTO and ATO, which might act as the n-type driving force in Ta-based codoping pairs [(Ta, Al) and (Ta, Ga)]. In the same group family with Ta, Nb had a similar electronic structure with Ta but an inferior mobility^36^ and thus might also play as the electron donor in Nb-based codoping pairs [(Nb, Al) and (Nb, Ga)]. In this way, it is inferable that Sr would be a rather strong electron acceptor in (Ta, Sr) and (Nb, Sr) pairs, resulting in an overall semiconducting or p-type conducting in codoping. These codoping effects for the pairs (Ta, Al), (Ta, Ga), (Nb, Al), and (Nb, Ga) have been experimentally validated as shown below.^37,38^ Provided that the mobility can be improved to be comparable with ITO, the cost of fabricating these alternative nonindium-based n-/p-type TCOs would be reduced. In addition, the flexibility of modulating electronic states would be increased, laying the foundation for manufacturing the cost-effective TCOs.

Our work focused more on the electronic states’ variations of SnO_2_ induced by preset codoping pairs. In the two-element codoping pairs, we have not only theoretically verified that codoping Ta, Nb, Ga, and Al can contribute to the conduction of SnO_2_ but also found that these conducting states can be tuned between n- and p-types by adjusting the doping concentrations of the donors (Ta or Nb) and the acceptors (Al or Ga). Especially for (Ta, Al) and (Nb, Al) pairs, p-type and n-type conducting metal oxides can be achieved just by adjusting the ratios of the element using similar targets or precursors. This will save the experimental time by eliminating the need of changing targets or precursors, thereby making the material processing more efficient. The codoped systems exhibited better tunability on the electronic states and accessibility of cheaper raw materials, which could be promising for practical applications in solar cells, displays, and transparent electronics. The potential of (Ta/Nb, Ga) pairs has been limited by the scarcity of Ga, which would cause a higher manufacturing cost (please see Figures S4 and S5 in the SI for the detailed electronic structures). The (Ta/Nb, Sr) pairs were prone to be tuned into p-type, which might lead to more complicated control parameters during processing for the appropriate electronic-state manipulations. Unlike the previous works on (Sb, Zn) and (Fe, Al) codopings,^25,39^ we have not calculated the specific electrical conductivity and optical transparency of these codoping pairs, which will require further studies in the future.

Table S1 summarizes the calculation results for three-element codoping for SnO_2_. These triplet combinations can be (Ta, Sr, V), (Ta, Sr, Nb), (Ta, Al, V), (Ta, Al, Nb), (Ta, Ga, V), and (Ta, Ga, Nb). Based on these calculations, we found that the electronic structures for (Ta, Sr, V) and (Ta, Sr, Nb) exhibit half-metal characteristics. It is the first time that magnetic TCOs have been demonstrated from first-principles calculations through three-element codoped SnO_2_. By comparing these six doping groups, we conjectured that the weaker electron negativity of Sr might enhance the spintronic properties of V and Nb, explaining the unobvious spin polarity in (Ta, Al, V), (Ta, Al, Nb), (Ta, Ga, V), and (Ta, Ga, Nb) groups. Although the optical properties were not discussed, the half-metal characteristics observed in (Ta, Sr, V/Nb) codoped SnO_2_ may also be useful to spintronic devices (please see Figure S6 for more details).

**Figure 3:**
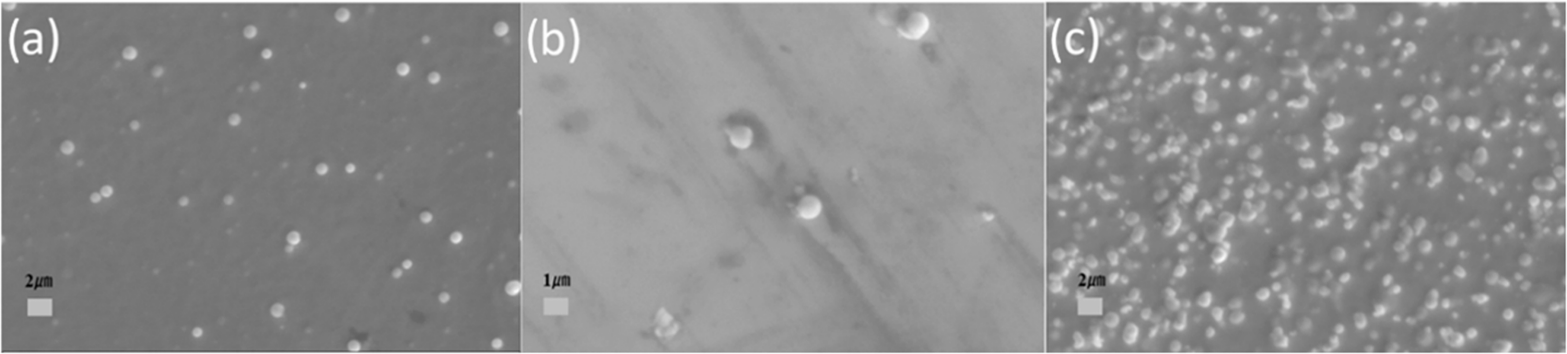
SEM images of Ta and Al codoped SnO_2_ thin films (TA1–3 in Table 3) with different substrate temperatures including (a) 350, (b) 400, and (c) 450 °C.

The codoped SnO_2_ thin films have been successfully deposited using the aerosol-assisted chemical vapor deposition method. Figure 3 illustrates how the substrate temperature affects the surface morphology of Ta–Al codoped SnO_2_ thin films. From the SEM images in Figure 3, it can be observed that at 350 °C (TA1, see Table 3), the surface of the deposited film was not very smooth, with large agglomerates on the surface of the films. At 400 °C (TA2), a compact thin film with a smooth surface was achieved. After the temperature was increased to 450 °C (TA3), larger agglomerated particles were formed due to the rapid evaporation of solvents before the aerosol precursor chemicals reached the heated substrate surface. The deposition temperature has a greater effect on the surface morphology than on the composition. The AFM images displayed in Figure S7 show that the deposited film is very smooth under a higher magnification. Although some agglomerates are present on the surface, they are primarily caused by a few large droplets during the spraying process. This could be improved by optimizing the deposition setup. In addition, the EDX mapping of the deposited film, as shown in Figure S8, indicates that the elemental distribution is quite uniform throughout the films.

The electrical properties of the deposited thin films are summarized in Table 3. As the deposition was processed in the air, the deposition temperature might affect the oxygen vacancy and the conductivity type of doped SnO_2_. When the precursor composition was 2.1 atom % for Ta and 4.2 atom % for Al, deposited at 350 and 400 °C, the p-type conductivity was observed. By contrast, the Ta–Al codoped SnO_2_ films using the same precursor ratio deposited at 450 °C exhibited the n-type conductivity, likely owing to an increase of the oxygen vacancies at the higher substrate temperature. At a deposition of 400 °C, consistent with our calculation results, a transition of the carrier polarity between the n-type and p-type occurring at the corresponding doping concentrations suggested by the calculations was observed when comparing TA2 and TA4. The sample TA2 with a doping concentration of 2.1 atom % for Ta and 4.2 atom % for Al exhibited an intense p-type conducting behavior, yielding a hole concentration of +2.030 × 10^18^ cm^–3^ (the positive sign refers to the hole transport). However, when the doping concentration reversed (4.2 atom % Ta and 2.1 atom % Al, TA4), the carrier polarity of the film was converted to n-type. By contrast, the conducting type of the sample TA4 is n-type with an electron concentration of −2.666 × 10^19^ cm^–3^ (the negative sign refers to the hole transport). The sheet resistance values of the TA thin films were approximately hundreds to thousands Ω/□. A further increase in the thickness of the deposited thin films would lead to a lower sheet resistance but also to a lower transmittance.

**Table 3:** Electrical Properties of Two-Element Codoped Groups and Concentrations of SnO_2_^a^

sample codoping SnO_2_: a, b	doping concentration (atom %)	thickness (nm)	resistivity (Ω·cm)	sheet resistance (Ω/□)	carrier concentration (cm^–3^)	mobility (cm^2^ V^–1^ s^–1^)
**TA1/350°C**	Ta (2.1%), Al (4.2%)	362	5.92 × 10^–4^	5.92 × 10^3^	+3.39 × 10^20^	0.179
**TA2/400°C**	Ta (2.1%), Al (4.2%)	249	1.30 × 10^–2^	525	+2.030 × 10^18^	185.5
**TA3/450°C**	Ta (2.1%), Al (4.2%)	314	1.41 × 10^–2^	450	–2.666 × 10^19^	3.467
**TA4/400°C**	Ta (4.2%), Al (2.1%)	376	6.31 × 10^–4^	1.67 × 10^3^	–2.52 × 10^19^	5.857
**NB1/350°C**	Nb (2.1%), Al (4.2%)	265	1.28 × 10^–1^	2.56 × 10^3^	+4.17 × 10^18^	11.69
**NB2/400°C**	Nb (2.1%), Al (4.2%)	167	1.21 × 10^–1^	6.069 × 10^3^	+2.45 × 10^18^	2.098
**NB3/450°C**	Nb (2.1%), Al (4.2%)	190	6.14 × 10^–2^	3.236 × 10^3^	+6.600 × 10^18^	14.62
**NB4/400°C**	Nb (4.2%), Al (2.1%)	300	2.27 × 10^–2^	4.73 × 10^3^	–4.26 × 10^19^	1.031
**NG/400 °C**	Nb (2.1%), Ga (4.2%)	185	2.96 × 10^–2^	1.602 × 10^3^	+1.133 × 10^19^	4.299
**TG/400 °C**	Ta (2.1%), Ga (4.2%)	204	3.21 × 10^–1^	15.74 × 10^3^	+3.268 × 10^19^	3.511

a For the carrier concentrations, the positive (negative) sign refers to hole (electron) transport.

As shown in Table 3, the type of conducting carriers in the Nb–Al codoped SnO_2_ samples was less influenced by the oxygen vacancies and the substrate temperature compared to Ta–Al codoped SnO_2_ when deposited in the air (NB1–4). All of the three codoped SnO_2_ films with the same precursor composition of 2.1 atom % for Nb and 4.2 atom % for Al but deposited at different substrate temperatures showed the p-type conductivity (NB1–3). To validate the Hall measurements of the conductive type in codoped SnO_2_, we also employed Seebeck measurements to confirm the carrier type. The Seebeck measurement results, as shown in Figure S9, further proved the p-type nature of codoped SnO_2_. We have also deposited Nb–Ga (NG), Ta–Ga (TG), and Nb–Al (NB2) codoped SnO_2_ (i.e., 2.1 atom % for Ta/Nb, 4.2 atom % for Ga/Al) at 400 °C, both of which demonstrated the p-type conductivity. In particular, we observed a transition between n- and p-type conductivities when reversing the relative doping concentration, as seen when comparing NB2 and NB4. From the summarized experimental results in Table 3, we can see that the Nb–Al pair shows the better consistency in terms of the n–p electronic property transitions, but they have a much higher sheet resistance compared to the Ta–Al pair, especially TA2. As shown in Table 3, the best p-type SnO_2_-based TCO we have synthesized here is the Ta–Al pair (2.1 atom % for Ta and 4.2 atom % for Al, TA2) deposited at 400 °C. To our best knowledge, the associated sheet resistance is in general comparable with that of Mg-doped LaCuOSe (∼275 Ω/□), the best-known p-type TCO.^41^

**Figure 4:**
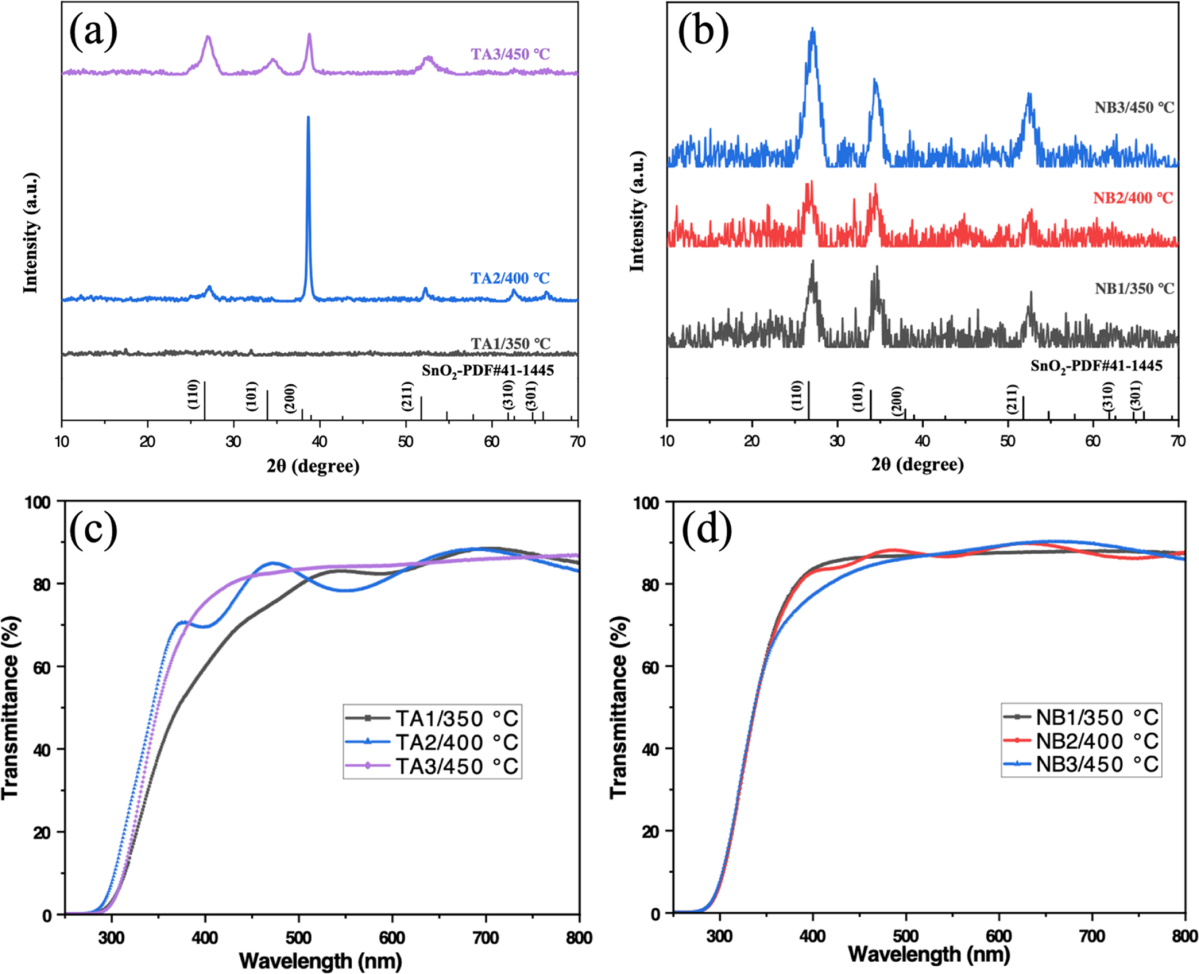
XRD patterns of (a) TA1–3 and (b) NB1–3. Optical transmittances of (c) TA1–3 and (d) NB1–3.

Figure 4 shows the XRD patterns of the crystalline phases in (a, b) and optical transmissions in (c, d) of the codoped SnO_2_ films, respectively. From Figure 4a,b, it can be seen that the codoped thin films show the characteristic peaks of SnO_2_. At a temperature above 400 °C, all of the codoped thin films are well crystallized. Both the Ta–Al codoped and Nb–Al codoped samples exhibited a high transmittance of above 80% in the visible wavelength ranging from 400 and 800 nm. Etaloning effects in the transmittance spectra of doped SnO_2_ thin films arise from the interference between light waves reflecting off the film’s interfaces. This interference creates the characteristic oscillations in the transmittance spectrum, which depend on factors such as the film’s thickness, refractive index, and surface quality. For the films with the same composition deposited at different substrate temperatures, the variations in etaloning effects are mainly due to the differences in the surface roughness. For the films of similar thicknesses, etaloning effects can also differ due to the variations in the chemical composition or the doping concentration (e.g., different levels of oxygen vacancies or doping elements). These variations can modify the refractive index and optical absorption, leading to a distinct etaloning behavior. Additionally, the changes in the material composition can affect the stress and crystal size (as shown in Table S2). This can alter the optical path length and interference conditions, thus further influencing the etaloning effects.

## Conclusions

In this report, we have performed: (1) a series of hybrid-exchange DFT calculations for two- and three-element codoped SnO_2_ and (2) a set of two-element codoped experiments accordingly to validate our calculations for the Ta–Al, Nb–Al, Nb–Ga, and Ta–Ga pairs. For two-element doping, we have computed the electronic structures of the pair formed by the donor (Ta/Nb) and the acceptor (Sr/Al/Ga) for the control of the n- and p-type electronic states. Especially for Al/Ga doping, the clear transitions between the n- and p-type electronic states have been manifested when varying the relative doping densities. By contrast, the electronic states would always be p-type for the Ta–Sr and Nb–Sr pairs.

To validate the modeling results, aerosol-assisted chemical vapor deposition, as a low-cost and easily scalable deposition method, was used to deposit the codoped SnO_2_ transparent conducting films with varied atomic ratios between dopants guided by the modeling. The deposition temperature of 400 °C resulted in a good crystallization of the codoped SnO_2_ transparent conducting films, with a high optical transmittance and good electrical conductivity. Notably, to our best knowledge, the p-type sheet resistance of TA2 is comparable to that of Mg-doped LaCuOSe, which has the lowest known sheet resistance.^40^ The sheet resistance can be further improved by optimizing the fabrication process, such as increasing the film thickness gradually. From the experimental results, the conductivity type of Ta–Al, Nb–Al, Nb–Ga, and Ta–Ga codoped SnO_2_ can be adjusted successfully by controlling the atomic ratio between V/III codopants in the precursors. For Ta–Al codoped films, the presence of O_2_ molecules in the air likely has a significant influence on the oxygen vacancy of the deposited films at a higher deposition temperature, thereby affecting the electrical properties. This requires a further detailed investigation. In addition, our codoping experimental strategy is highly efficient because we use the same target materials/precursors during material processing. More importantly, based on our work, we can prevent the lattice mismatching when fabricating transparent p–n junctions, which is a clear advantage over using two different types of host materials. Moreover, when transition metal atoms were used in the three-element codoping, the resulting electronic states could be half-metallic, providing spin-polarized electrical current, thus leading to magnetic TCOs that combine magnetic properties with those of TCOs.

In summary, our work has provided a solid foundation for the design, fabrication, and control of the n- and p-type TCOs, in addition to the magnetic TCOs. Importantly, we have also validated our simulation results experimentally, thus pointing to combining first-principles material design and experimentally codoping SnO_2_ to fabricate bespoke TCOs. In the future, we would like to investigate more doping elements (e.g., the group II and III elements) or different doping densities to enhance the electrical conductivity. We could also integrate the machine learning technique to accelerate the material discovery process.

## Data Availability

The data underlying this study are not publicly available due to the proprietary nature and institutional constraints. All computer codes and data that support the findings of this study are available from the corresponding author upon reasonable request.

## Supporting Information Available

Computed band structure and density of states for Ta/Nb–Sr and Ta/Nb–Ga dopant pairs; computed band structures for three-element codoping Ta–Sr–V and Ta–Sr–Nb; computational results for three-element codoping (Table S1); experimental results including 2D AFM images for the Ta–Al codoped sample; EDX spectra and elemental mapping for the Nb–Ga codoped sample; Seebeck results for the Nb–Al codoped sample; and crystal sizes for a variety of codoped samples (Table S2) (PDF)
