# Supplementary material for: Tunable Transparent Conductors Based on SnO2: Theoretical and Experimental Studies of Codoping
Source: ACS Omega. 2024 Dec 4;9(50):49674–82. doi: 10.1021/acsomega.4c07860 (PMC11656248; doi:10.1021/acsomega.4c07860)
Supplement: Supplementary file 1 — ao4c07860_si_001.pdf [file ao4c07860_si_001.pdf]

Tunable transparent conductors based on SnO_2: Theoretical and experimental studies of co-doping

Wenjing Qian#, Xianghui Feng#, Yanxue Wang#, Ahmet Nazligul, Yiwen Lu, Mingqing Wang*, Wei Wu*[1] and Kwang Leong Choy*[2]

UCL Institute for Materials Discovery, University College London, Malet Place, London WC1E 7JE, United Kingdom.

*Corresponding emails: mingqing.wang@ucl.ac.uk, wei.wu@ucl.ac.uk, kwang.choy@duke.edu
#These authors contributed equally

[1] Current Address: Beijing National Laboratory for Condensed Matter Physics, Institute of Physics, Chinese Academy of Sciences, Beijing 100190, China
[2] Current Address: Duke Kunshan University, Division of Natural and Applied Sciences, Jiangsu, China.

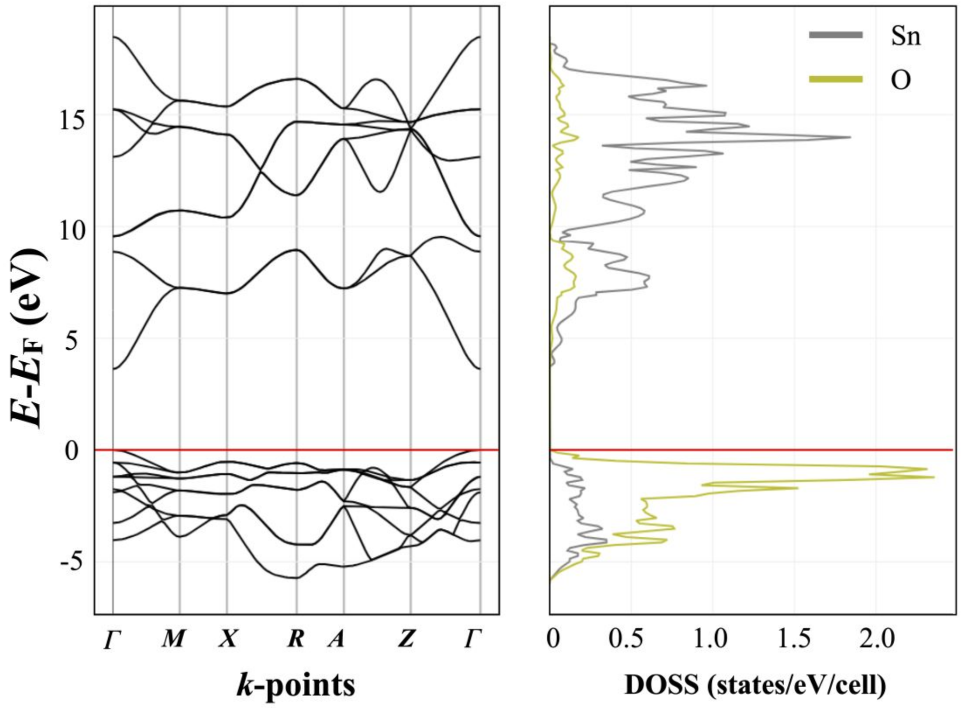

Figure S1: The band structure (a) and the density of states (DOSS) of the pure rutile SnO_2.

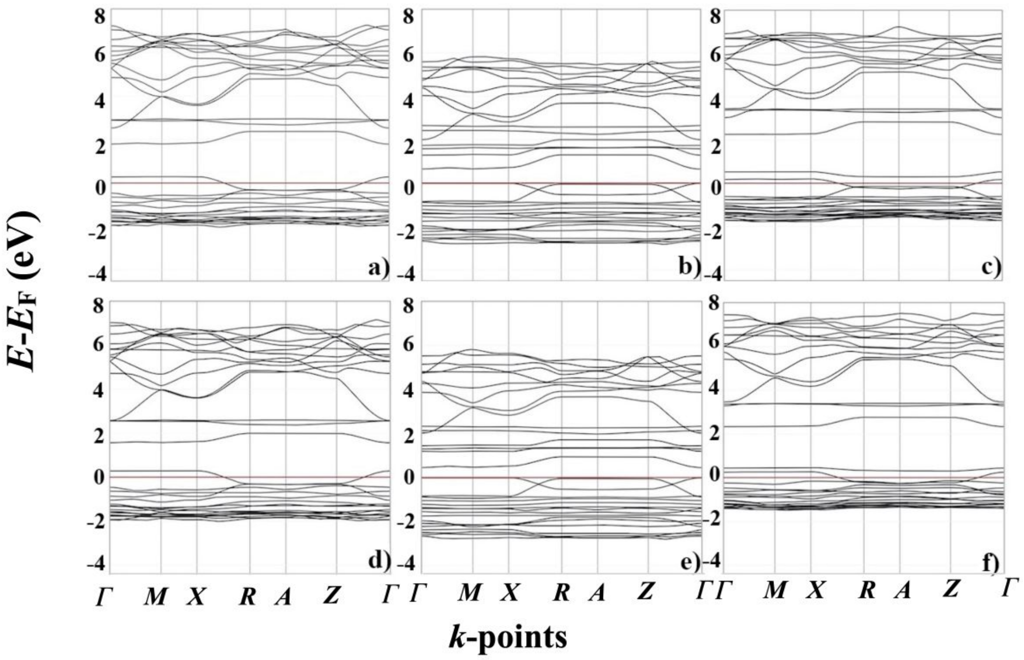

Figure S2: The band structures of: a) 1 Ta atom and 1 Sr atom per cell; b) 2 Ta atoms and 1 Sr atom per cell; c) 1 Ta atom and 2 Sr atoms per cell; d) 1 Nb atom and 1 Sr atom per cell; e) 2 Nb atoms and 1 Sr atom per cell; and f) 1 Nb atom and 2 Sr atoms per cell for the co-doped SnO_2.

As shown in Figure S2, we present the band structures for Ta-Sr co-doped SnO_2, where the effects of co-doping Ta and Sr are visible. The electronic state can be tuned by adjusting the composition of the n- and p-type dopants. As we can see from Figure S2(a), when co-doping SnO_2 supercell with 1 Ta atom/cell and 1 Sr atom/cell (corresponding to 2.1% doping), this would result in a p-type conducting state. By contrast, the co-doping with 2 Ta atoms/cell and 1 Sr atom/cell would result in a semiconducting state with a very small band gap (~0.6 eV). The co-doping with 1 Ta atom/cell and 2 Sr atoms/cell would lead to a p-type conducting state with deeper Fermi energy level with reference to the valence band maximum (VBM). Such similar behaviour has been observed in the co-doping between Nb and Sr atoms as shown in Figure S2 (d-f) in the main text.

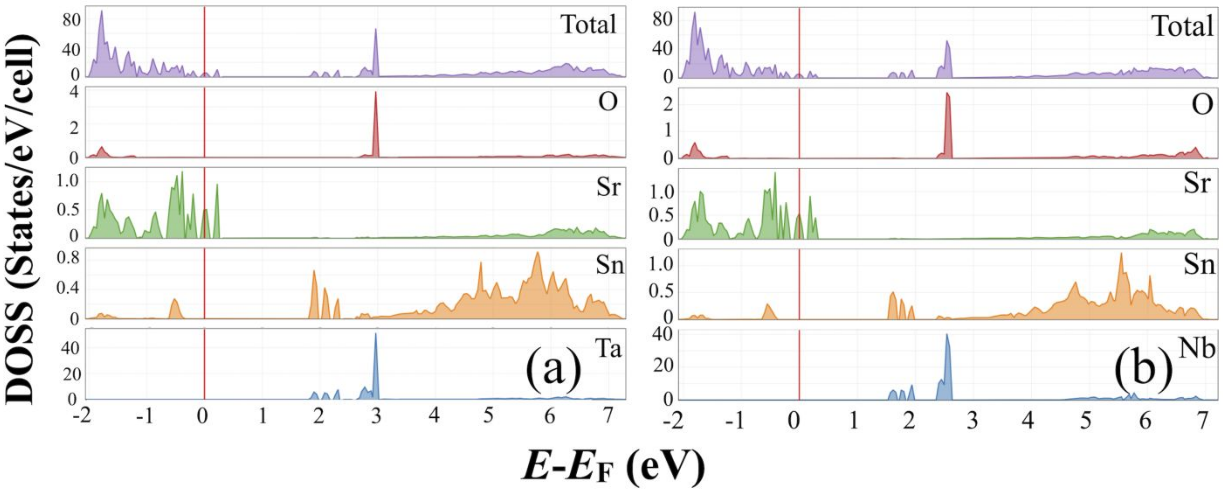

Figure S3: Density of states graphs of: a) 1 Ta and 1 Sr per cell; and b) 1 Nb and 1 Sr per cell for the co-doped SnO_2.

In Figure S3, we can observe the PDOS for Ta-Sr and Nb-Sr co-doped SnO_2, which suggests the contributions from different atoms to the band structure. As shown in Figure S3(a) and (b), the Sr atomic orbitals are dominant at the Fermi energy (below the VBM) in both cases, indicating the importance of Sr atoms in the p-type electronic state. By contrast, the conduction band minimum (CBM) is mainly composed of Sn and Ta (Nb) atomic orbitals. We have explored these results and provided guidance for the experimental design of the p-type TCOs.

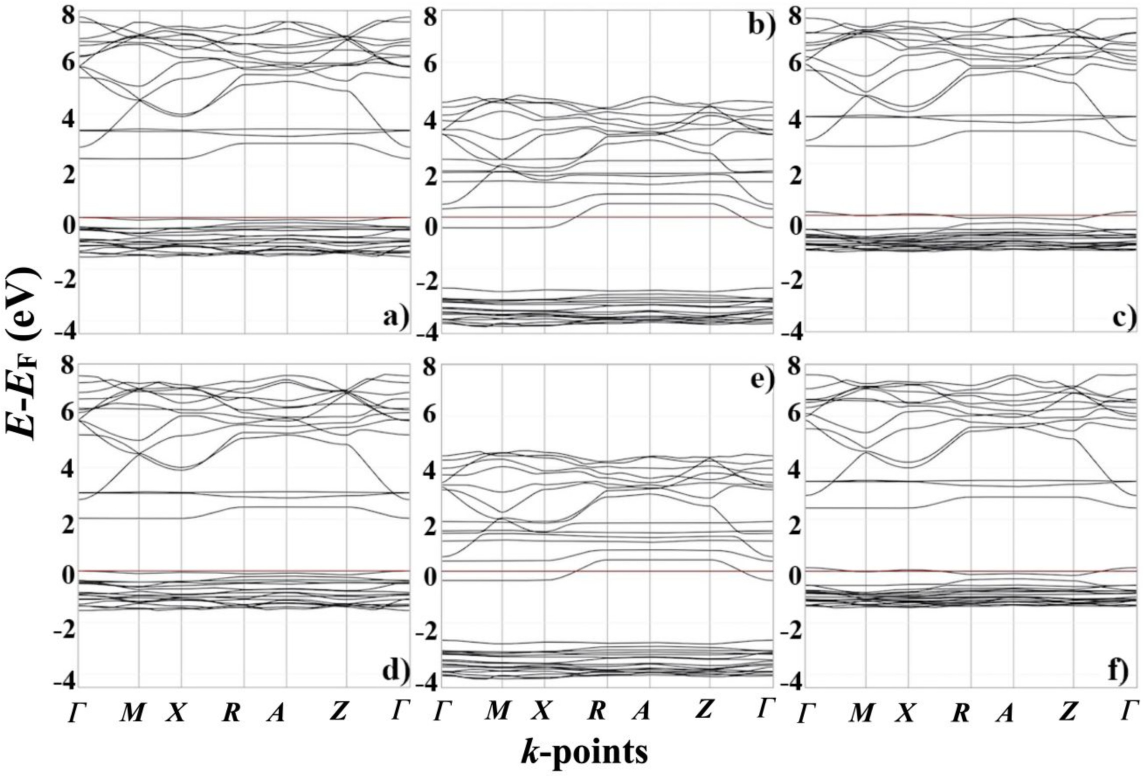

Figure S4: The band structures of theco-doped SnO_2 with: a) 1 Ta atom/cell and 1 Ga atom/cell; b) 2 Ta atoms/cell and 1 Ga atom/cell; c) 1 Ta atom/cell and 2 Ga atoms/cell; d) 1 Nb atom/cell and 1 Ga atom/cell; e) 2 Nb atoms/cell and 1 Ga atom/cell; f) 1 Nb atom/cell and 2 Ga atoms/cell.

The co-doping of Ta and Ga in SnO_2 (see Figure S4) exhibits a similar tuning process as the co-doping of Ta and Al. As shown in Figure S5, the electronic density at the Fermi energy is dominated by the Ga atom, whereas at the CBM, the Sn and Ta atoms make significant contributions to n-type charge carriers.

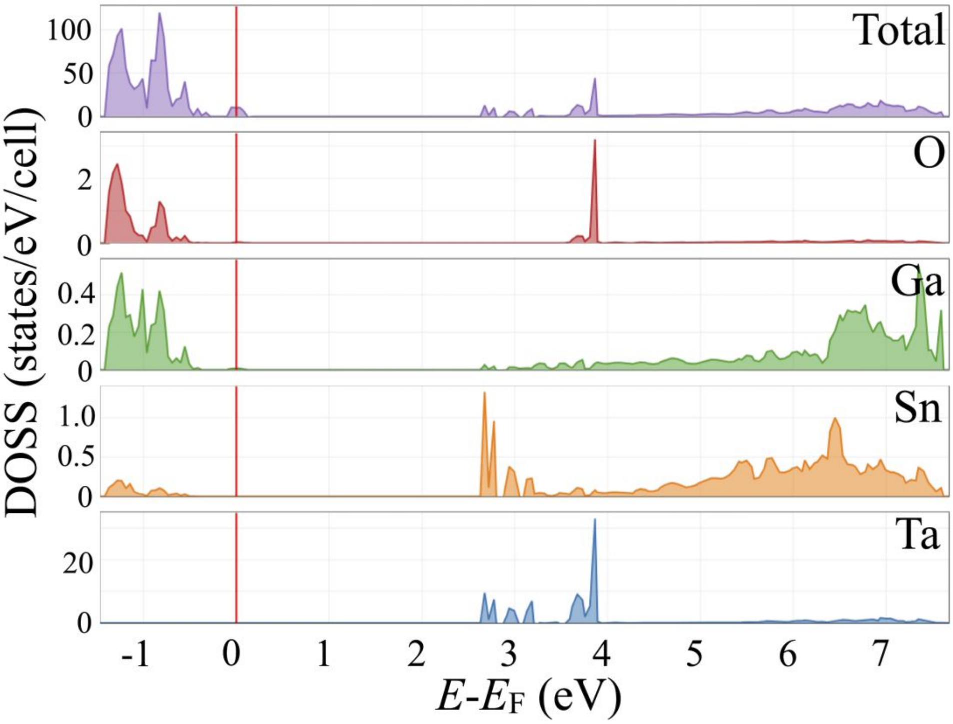

Figure S5: Density of states graphs of 1 Ta atom/cell and 2 Ga atoms/cell in co-doped SnO_2.

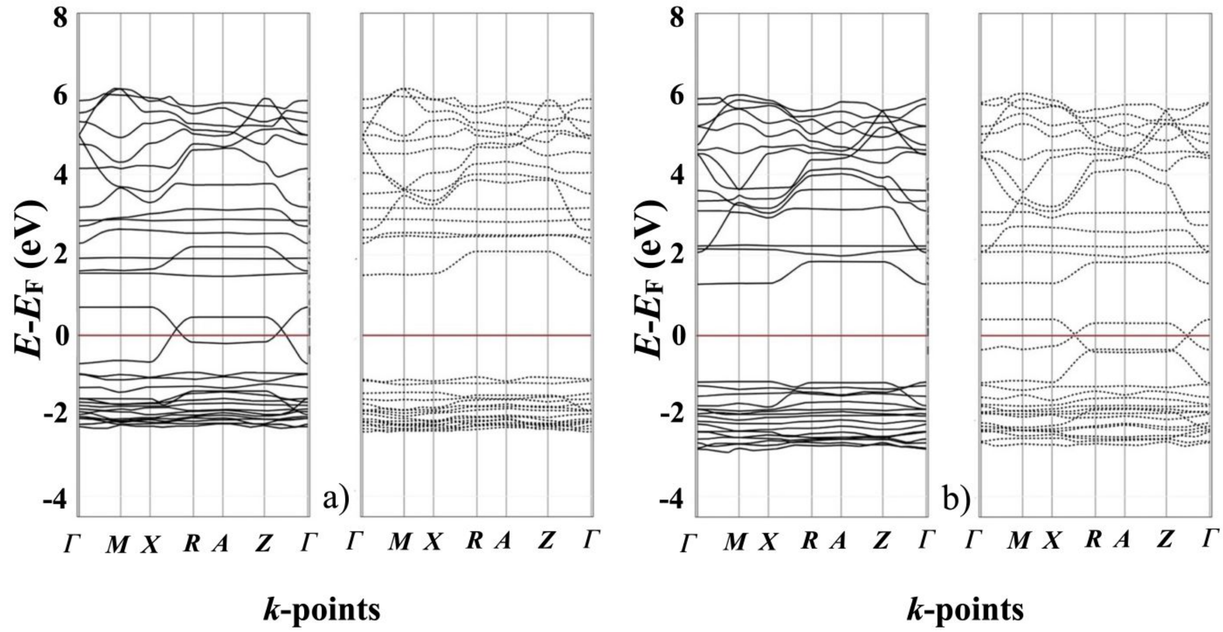

Figure S6: The open-shell calculations for the band structures of the co-doped SnO_2 with (a) one Ta atom, one Sr atom and one V atom per cell and (b) one Ta atom, one Sr atom and one Nb atom per cell.

Figure S6 shows the co-doping of three elements including Ta, Sr, and V (Nb). As V is a transition metal, here we have performed the open-shell calculations to illustrate the effect of spins. We can see that both cases (Ta and Nb) exhibited the interesting half-metal characteristics for the electronic state (one spin channel conducting while the other insulating or semiconducting). This points to the concept of magnetic TCO, which could be useful for future spintronic devices. The local spin of the *d*-orbital electron in vanadium has been reported to induce ferromagnetism in V-doped SnO_2[37] and ZnO[38], which is the origin of half-metal characteristics in (Ta, Sr, V) co-doping. Similarly, the conducting state in (Ta, Sr, Nb) co-doping might stem from the 4*d*-orbital of Nb.

Table S1. Three-element co-doped groups and concentrations of SnO_2

Co-doping	Doping Concentrations (at. %)	Band structure (spin up)	Band structure (spin down)
Ta, Sr, and V	Ta (2.1%), Sr (2.1%), V (2.1%)	Conducting	Semiconducting (2.58 eV)
Ta, Sr, and Nb	Ta (2.1%), Sr (2.1%), Nb (2.1%)	Semiconducting (2.43 eV)	Conducting
Ta, Al, and V	Ta (2.1%), Al (2.1%), V (2.1%)	Semiconducting (2.30 eV)	Semiconducting (2.13 eV)
Ta, Al, and Nb	Ta (2.1%), Al (2.1%), Nb (2.1%)	Semiconducting (1.56 eV)	Semiconducting (2.55 eV)
Ta, Ga, and V	Ta (2.1%), Ga (2.1%), V (2.1%)	Semiconducting (2.41 eV)	Semiconducting (2.42 eV)
Ta, Ga and Nb	Ta (2.1%), Ga (2.1%), Nb (2.1%)	Semiconducting (1.56 eV)	Semiconducting (2.59 eV)

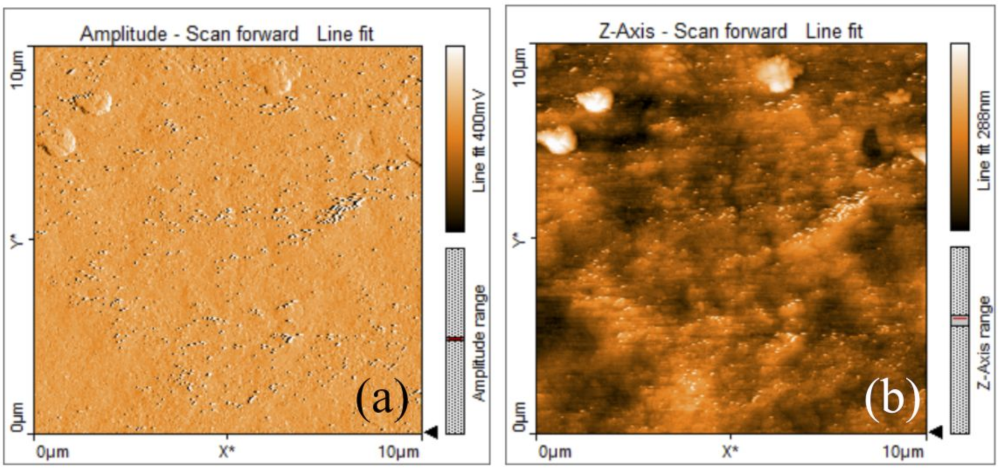

Figure S7. The 2D AFM phase image (a) and height image (b) of the aerosol assisted chemical vapor deposition of the Ta(2.1at%) and Al(4.2at%) co-doped SnO_2 film at 400°C.

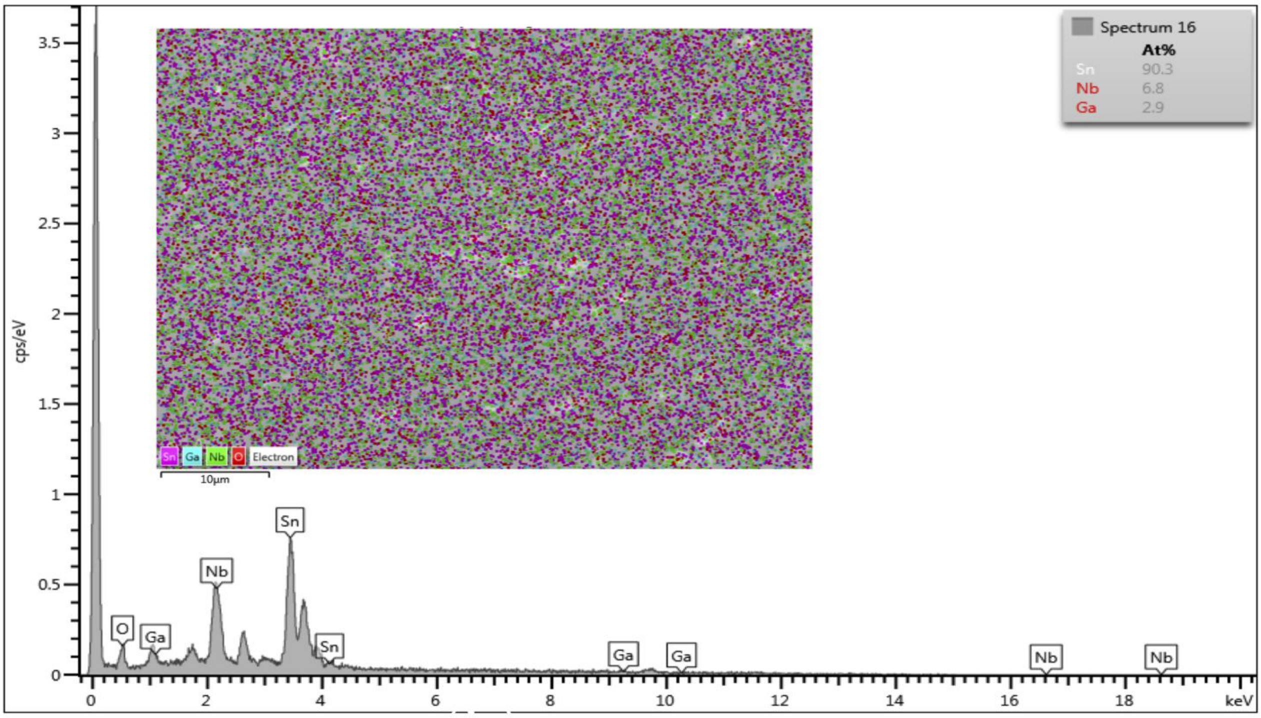

Figure S8: The EDX spectra of Nb-Ga co-doped SnO_2 film (the sample NG listed in Table 1 of the main text) is shown. The inset is the EDX mapping.

To fortify the accuracy of our findings, we employed the Seebeck measurements in conjunction with the Hall effect. The results from these measurements were in a good agreement, as demonstrated in Figure S9. Specifically, samples NA manifested positive Seebeck coefficients, signalling their p-type nature.

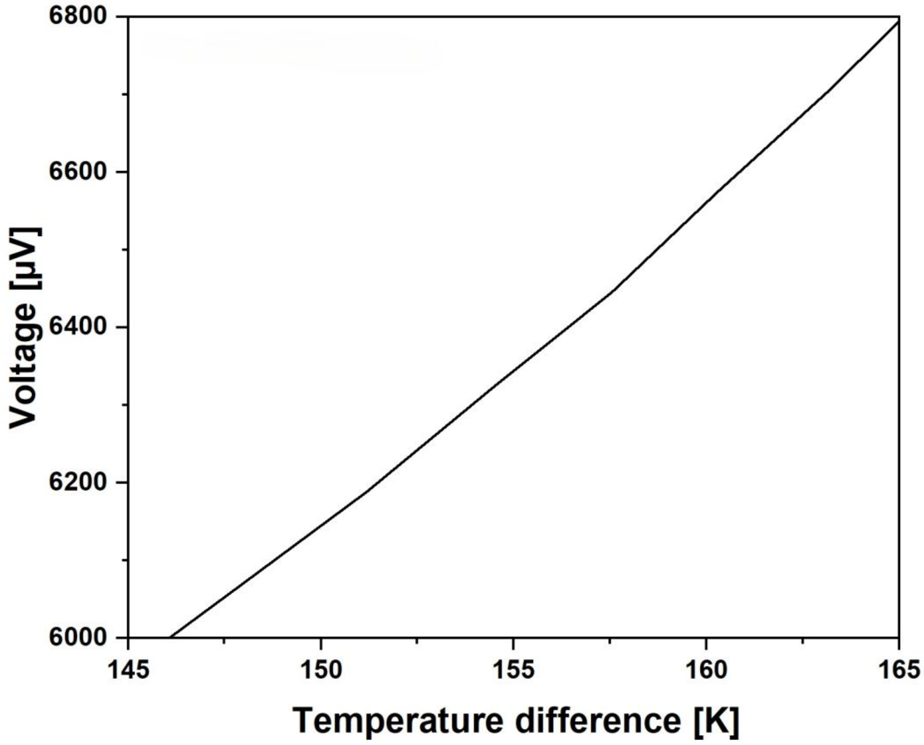

Figure S9: The Seebeck voltage of the NA sample listed in Table.1 of the main text, as a function of the temperature.

Utilising the Scherrer formula, the film's grain sizes were determined via $D = \frac{0.9\lambda}{\beta \cos\theta}$. Here D represents the nanoparticle's particle size, β signifies the full width at half maximum (FWHM) of the peak in radians, and θ denotes the Bragg angle. The grain size values are tabulated in Table S2.

Table S2: Crystal size of varied doped SnO_2 films deposited at 400°C

Sample	Crystal size (nm)
TA2	10.41
TA4	10.62
NB2	25.17
NB4	25.32

From Table S2, it can be seen the dopant concentration affects the crystal size of the deposited thin films. For Ta and Al co-doped samples, the increased content of Ta leads to slightly increased crystal

size. Compared with Ta and Al coped samples, the Nb and Ga co-doped samples show bigger crystal size. This might be due to the smaller cation size difference between Nb^{5+} (0.69nm),Ga^{3+} (0.062nm) and Sn^{4+} (0.69nm) compared with that between Ta^{5+} (0.073nm) and Al^{3+} (0.053nm).

References

1. Dixon, S. C., Scanlon, D. O., Carmalt, C. J. & Parkin, I. P. N-Type doped transparent conducting binary oxides: An overview. *J. Mater. Chem. C* **4**, 6946–6961 (2016).
2. Lewis, B. G. & Paine, D. C. Applications and processing of transparent conducting oxides. *MRS Bull.* **25**, 22–27 (2000).
3. Fortunato, E., Ginley, D., Hosono, H. & Paine, D. C. Transparent conducting oxides for photovoltaics. *MRS Bull.* **32**, 242–247 (2007).
4. Liu, H., Avrutin, V., Izyumskaya, N., Özgr, Ü. & Morkoç, H. Transparent conducting oxides for electrode applications in light emitting and absorbing devices. *Superlattices and Microstructures* vol. 48 458–484 (2010).
5. Ma, C. H., Chen, E. L., Lai, Y. H., Chen, Y. C., Chang, L. and Chu, Y. H., Flexible transparent heteroepitaxial conducting oxide with mobility exceeding 100 cm2 V^{-1} s^{-1} at room temperature. *NPG Asia Mater.* **12**, 70 (2020).
6. Sathyamoorthy, R., Abhirami, K. M., Gokul, B., Gautam S., Chae, K. H., & Asokan, K. Fabrication of p-n junction diode using SnO/SnO_2 thin films and its device characteristics. *Electron. Mater. Lett.* **10**, 743–747 (2014).
7. Zhang, K. H. L., Xi, K., Blamire, M. G. & Egdell, R. G. P-type transparent conducting oxides. *J. Phys. Condens. Matter* **28**, 383002 (2016).
8. Batzill, M. & Diebold, U. The surface and materials science of tin oxide. *Prog. Surf. Sci.* **79**, 47–154 (2005).
9. Kim, H., Auyeung, R. C. Y. & Piqué, A. Transparent conducting F-doped SnO_2 thin films grown by pulsed laser deposition. *Thin Solid Films* **516**, 5052–5056 (2008).
10. Maleki, M., Electronic structure and optical properties of nitrogen doped SnO_2 - Simulation by DFT method. *Acta Phys. Pol. A* **137**, 272–275 (2020).
11. Behtash, M., Joo, P. H., Nazir, S. & Yang, K. Electronic structures and formation energies of pentavalent-ion-doped SnO_2: First-principles hybrid functional calculations. *J. Appl. Phys.* **117**, (2015).
12. Hu, Y., Li, L., Xu, C. & Yang, P. Study of high metal doped SnO_2 for photovoltaic devices. *Mater. Today Commun.* **27**, 102148 (2021).

13. Nakao, S., Yamada, N., Hitosugi, T., Hirose, Y., Shimada, T. and Hasegawa, T.,. High mobility exceeding 80 cm^2 V^{-1} s^{-1} in polycrystalline Ta-Doped SnO_2 thin films on glass using anatase TiO_2 seed layers. *Appl. Phys. Express* **3**, 031102 (2010).
14. Ramarajan, R., Kovendhan, M., Babu, R. R., Thangaraju, K. & Joseph, D. P. Optimization and transport properties of 'Nb' doped SnO_2 thin film as an alternate TCO application. *AIP Conf. Proc.* **2115**, 2–6 (2019).
15. Medina Chanduvi, H.H., Mudarra Navarro, A.M., Bilovol, V., Errico, L.A. and Gil Rebaza, A.V., 2021. Structural, Electronic, Magnetic, and Hyperfine Properties of V-doped SnO2 (Sn1–x V x O2, x: 0, 0.042, 0.084, and 0.125). A DFT-Based Study. *The Journal of Physical Chemistry C* **125**, 11702-11713 (2021).
16. Tsay, C. Y. & Liang, S. C. Fabrication of p-type conductivity in SnO_2 thin films through Ga doping. *J. Alloys Compd.* **622**, 644–650 (2015).
17. Yang, Y., Zhou, W., Liang, Y., Liu, L. & Wu, P. Tuning band gap and ferromagnetism in epitaxial Al-doped SnO_2 films by defect engineering. *J. Cryst. Growth* **430**, 75–79 (2015).
18. Hunashimarad, B. G., Bhat, J. S., Raghavendra, P. V. & Bhajantri, R. F. Photoluminescence in Strontium doped tin oxide thin films. *Opt. Mater. (Amst).* **114**, 110962 (2021).
19. Swallow, J. E. N., Williamson, B.A., Whittles, T.J., Birkett, M., Featherstone, T.J., Peng, N., Abbott, A., Farnworth, M., Cheetham, K.J., Warren, P. and Scanlon, D.O., *Adv. Funct. Mater.* **28**, 1701900 (2018).
20. Ponja, S. D., Williamson, B. A., Sathasivam, S., Scanlon, D. O., Parkin, I. P. and Carmalt, C. J.,, Enhanced electrical properties of antimony doped tin oxide thin films deposited: Via aerosol assisted chemical vapour deposition. *J. Mater. Chem. C* **6**, 7257–7266 (2018).
21. Williamson, B. A. D. *et al.* Resonant Ta Doping for Enhanced Mobility in Transparent Conducting SnO_2. *Chem. Mater.* **32**, 1964–1973 (2020).
22. Lv, S., Zhou, Y., Xu, W., Mao, W., Wang, L., Liu, Y. and He, C., Preparation of p-type GaN-doped SnO_2 thin films by e-beam evaporation and their applications in p–n junction. *Appl. Surf. Sci.* **427**, 64–68 (2018).
23. Moharrami, F., Bagheri-Mohagheghi, M. M. & Azimi-Juybari, H. Study of structural, electrical, optical, thermoelectric and photoconductive properties of S and Al co-doped SnO_2 semiconductor thin films prepared by spray pyrolysis. *Thin Solid Films* **520**, 6503–6509 (2012).
24. Dang, H. P., Luc, Q. H., Nguyen, T. T. & Le, T. Eliminating the charge compensation effect in Ga-doped SnO_2 films by N doping. *J. Alloys Compd.* **776**, 276–286 (2019).
25. Saadeddin, I., Pecquenard, B., Manaud, J.P., Decourt, R., Labrugère, C., Buffeteau, T. and Campet, G., Synthesis and characterization of single- and co-doped SnO_2 thin films for

optoelectronic applications. *Appl. Surf. Sci.* **253**, 5240–5249 (2007).

26. Venkateswara Reddy, P., Venkatramana Reddy, S. & Sankara Reddy, B. Synthesis and properties of (Fe, Al) co-doped SnO_2 nanoparticles. *Mater. Today Proc.* **3**, 1752–1761 (2016).
27. Zhou, L., Wang,J., Chen, L., Yu, S., Zhu, Y.. First principles study on the electrical conductivity of S and La co-doped SnO_2 . *Precious Metals* 8 , 64-71 (2019).
28. Dovesi, R., Orlando, R., Civalleri, B., Roetti, C., Saunders, V. R. and Zicovich-Wilson, C. M., CRYSTAL: A computational tool for the ab initio study of the electronic properties of crystals. *Zeitschrift fur Krist.* **220**, 571–573 (2005).
29. Zinola, C. F. Density functional theory. *Electrocatal. Comput. Exp. Ind. Asp.* 117–138 (2010).
30. Baur, W. H. & Khan, A. A. Rutile-type compounds. IV. SiO_2, GeO_2 and a comparison with other rutile-type structures. *Acta Crystallogr. Sect. B Struct. Crystallogr. Cryst. Chem.* **27**, 2133–2139 (1971).
31. Hou, X. & Choy, K. L. Processing and applications of aerosol-assisted chemical vapor deposition. *Chem. Vap. Depos.* **12**, 583–596 (2006).
32. Smits, F. M. Measurement of Sheet Resistivities with the Four-Point Probe. *Bell Syst. Tech. J.* **37**, 711–718 (1958).
33. On a New Action of the Magnet on Electric Currents, E . H . Hall, The Johns Hopkins University Press.
34. Shao, T., Zhang, F. & Zhang, W. Density functional theory study on the electronic structure and optical properties of SnO_2. *Rare Met. Mater. Eng.* **44**, 2409–2414 (2015).
35. Barbarat, P. & Matar, S. F. First-principles investigations of the electronic, optical and chemical bonding properties of SnO_2. *Comput. Mater. Sci.* **10**, 368–372 (1998).
36. Yude Wang, Torsten Brezesinski, Markus Antonietti, and Bernd Smarsly, Ordered Mesoporous Sb-, Nb-, and Ta-Doped SnO_2 Thin Films with Adjustable Doping Levels and High Electrical Conductivity. 1373–1378 (2009).
37. Popa, A., Toloman, D., Raita, O., Stan, M., Pana, O., Silipas, T. D. and Giurgiu, L. M., Ferromagnetic behaviour of vanadium doped SnO_2 nanoparticles annealed at different temperatures. *J. Alloys Compd.* **591**, 201–206 (2014).
38. Jiang, F. X., Tong, R. X., Yan, Z., Ji, L. F. & Xu, X. H. d-electron-dependent transparent conducting oxide of V-doped ZnO thin films. *J. Alloys Compd.* **822**, 153706 (2020).
39. Saadeddin, I., Hilal, H.S., Pecquenard, B., Marcus, J., Mansouri, A., Labrugère, C., Subramanian, M.A. and Campet, Simultaneous doping of Zn and Sb in SnO_2 ceramics: Enhancement of electrical conductivity. *Solid State Sci.* **8**, 7–13 (2006).

40. Willis, J. & Scanlon, D. O. Latest directions in p-type transparent conductor design. *J. Mater. Chem. C* **9**, 11995–12009 (2021).
41. H. Hiramatsu, K. Ueda, H. Ohta, M. Hirano, T. Kamiya and H. Hosono, Degenerate *p*-type conductivity in wide-gap $LaCuOS_{1-x}Se_x$ (x=0-1) epitaxial films, Appl. Phys. Lett. 82 (7), 1048-1050 (2003).
